# Smoking increases risks of all-cause and breast cancer specific mortality in breast cancer individuals: a dose-response meta-analysis of prospective cohort studies involving 39725 breast cancer cases

**DOI:** 10.18632/oncotarget.13366

**Published:** 2016-11-15

**Authors:** Kang Wang, Feng Li, Xiang Zhang, Zhuyue Li, Hongyuan Li

**Affiliations:** ^1^ Department of Endocrine and Breast Surgery, The First Affiliated Hospital of Chongqing Medical University, Chongqing, 400016, China; ^2^ Department of Neurosurgery, The Second Affiliated Hospital of Chongqing Medical University, Chongqing, 400010, China; ^3^ Department of Nursing, The First Affiliated Hospital of Chongqing Medical University, Chongqing, 400016, China

**Keywords:** smoking, breast cancer survival, dose-response, all-cause mortality, breast cancer specific mortality

## Abstract

Smoking is associated with the risks of mortality from breast cancer (BC) or all causes in BC survivors. Two-stage dose-response meta-analysis was conducted. A search of PubMed and Embase was performed, and a random-effect model was used to yield summary hazard ratios (HRs). Eleven prospective cohort studies were included. The summary HR per 10 cigarettes/day, 10 pack-years, 10 years increase were 1.10 (95% confidence interval (CI) = 1.04–1.16), 1.09 (95% CI = 1.06–1.12), 1.10 (95% CI = 1.06–1.14) for BC specific mortality, and 1.15 (95% CI = 1.10–1.19), 1.15 (95% CI = 1.10–1.20), 1.17 (95% CI = 1.11–1.23) for all-cause mortality, respectively. The linear or non-linear associations between smoking and risks of mortality from BC or all causes were revealed. Subgroup analyses suggested a positive association between ever or former smoking and the risk of all-cause mortality in BC patients, especially in high doses consumption. In conclusion, higher smoking intensity, more cumulative amount of cigarettes consumption and longer time for smoking is associated with elevated risk of mortality from BC and all causes in BC individuals. The results regarding smoking cessation and “ever or former” smokers should be treated with caution due to limited studies.

## INTRODUCTION

Breast cancer (BC) has become the most common cancer among women, accounting for approximately 29% all new cancer diagnoses worldwide [1]. Although BC is the most frequent cause of cancer death in women in less developed regions [2] and BC specific mortality takes great proportion especially in late BC survivors [3], the average 5-year cause-specific survival exceeds the 90% [4]. Long time BC survival is so common that BC survivors represents 41% female cancer survivors in America [5]. To achieve a reasonable surveillance for this large scale BC survivors, Guideline from the American Cancer Society (ACS) BC Survivorship Care recommended various primary cares, emphasizing the importance of death prevention through corrected clinical decisions and modifiable related lifestyle factors [6]. Among these lifestyle factors, normal weight [7] and physical activity [8] had been suggested to reduce the risk for BC specific and for all-cause death in BC survivors.

Smoking is responsible for approximately 10% to 12% BC survivors [9, 10]. Guidelines from ACS BC Survivorship Care suggested that primary care clinicians should counsel BC survivors to avoid smoking [6], and a meta-analysis [11] conducted in 2014 indicated that compared to non-smokers, the BC survivors in current smoking were at increased risk of BC-specific mortality. However, high heterogeneity was found among included studies, raising concerns about the reliability of its summary results. Mounting epidemiological studies were conducted to further investigate the association of smoking doses or duration and risks of BC specific and all-cause mortality [11–21], but they presented inconsistent results. Some studies observed that high intensity (> 30 cigarettes/day), high cumulative amount of cigarettes (> 30 pack-years), long duration of smoking (> 20 years) or short time since quitting smoking (< 5 years) increased the risks of mortality from all cause and BC in subjects with BC [12, 13, 15–17], whereas others failed to find any significant associations [14, 20, 21]. Additionally, a recent cohort study indicated that lifetime smoking exposure, rather than smoking status, should be used to assess mortality risk among BC survivors [19], and a series of results from epidemiological studies [13, 14, 16, 18] supported this point, showing that elevated risk of mortality in BC individuals was also observed among former smokers when they had inhaled a large amount of cigarettes before. A previous meta-analysis suggested a linear pattern for the association between duration of ever active smoking and BC incidence [22]. Whether the linear pattern can be extended to the association of smoking in BC survivors and risks of mortality from all causes and BC is largely unknown.

With arousing considerable attention to the survival of BC, investigation dose-response relationship between smoking and risks of all-cause mortality and BC specific mortality is critical for a better understanding of this important lifestyle factor in BC survival. Up to date, to the best of our knowledge, no dose-response meta-analysis on this topic is available. Therefore, the objectives of our study were to reveal the dose-response associations between various smoking dimensions (i.e. intensity, cumulative amount of cigarettes, duration and cessation) and risks of mortality from all causes and BC in subjects with BC, and to further investigate the precise shape of these associations.

## RESULTS

### Study selection

**Figure 1:**
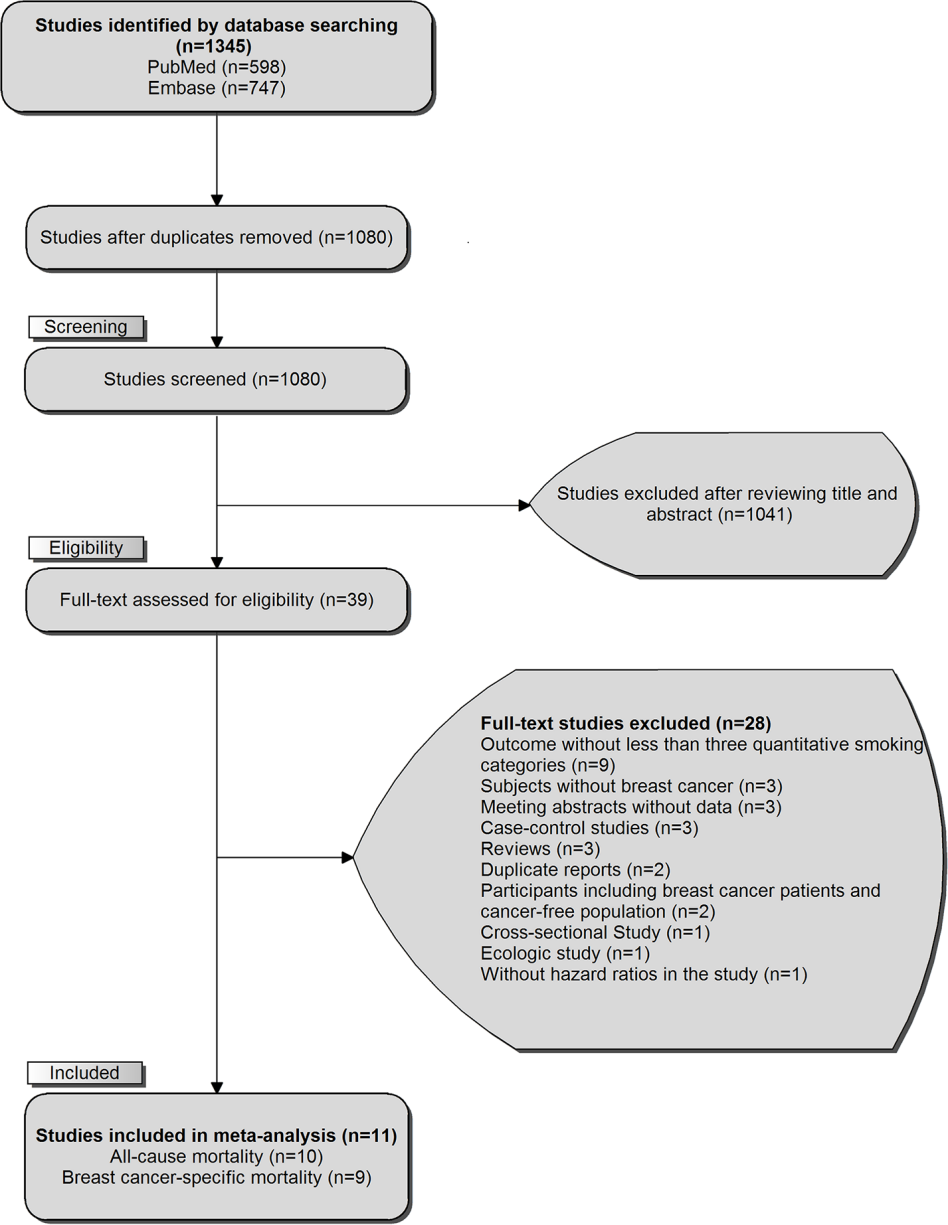
The flowchart of selecting eligible studies

The comprehensive literature research and selection identified 598 articles and 747 articles from PubMed and EMBASE, respectively. After removing 265 duplicates, we reviewed the titles and abstracts of 1080 articles, and 1041 obvious irrelevant citations were excluded. The remaining 39 articles were assessed in more detail for eligibility by reading the full text. Among them, 28 were excluded (detailed reasons for exclusion are shown in Supplementary Table S1). In the last, 11 prospective cohort studies were used for final data synthesis. The flow chart of literature searching was presented in Figure 1.

### Study characteristics

The characteristics of 11 eligible studies are presented in Table 1. These studies were published from 2007 [21] to 2016 [12, 13]. The associations between smoking intensity, cumulative amount of cigarettes, smoking duration, smoking cessation and risk of BC specific mortality were reported by 7 [11, 14, 16, 17, 19–21], 9 [11, 12, 14–19, 21], 6 [11, 12, 14, 16, 19, 21], and 2 [12, 17] studies, respectively. And 7 [11, 14, 16, 17, 19–21], 9 [11–14, 16–19, 21], 6 [11, 12, 14, 16, 19, 21], 2 [12, 17] studies referred to associations between these smoking dimensions and the risk of all-cause mortality, respectively. Two studies were conducted in Europe [17, 20], 11 in America [11, 12, 15, 16, 18, 19, 21], 1 in Asia [14], and the remaining a transnational study in America and Asia [13]. The mean age of participants ranged from 54.5 [16] to 63 [15] across studies, and the sample size (BC cases) varied from 843 [14] to 6596 [13]. This meta-analysis included 39725 BC cases, and they are all female. The followed-up duration changed from 5.6 years [21] to 12.0 years [13]. After 357525 person-years following, 11654 subjects died in total, consisting of 7647 for all causes, and 4007 for BC specific cause. Smoking statuses were ascertained after diagnosis of BC in most studies [11–16, 18, 21], whereas one study addressed them before diagnosis [17]. Smoking status varied cross studies, most studies [11, 12, 15–17, 20, 21] included subjects who were on current smoking, some studies [14, 16, 19, 21] enrolled subjects on ever smoking, and only two studies [13, 18] reported associations on former smoking and risks of death from all causes and BC in BC survivors. Current smoking was defined if BC patients smoked at time of interview, while Kakugawa et al [14] and Seibold et al. [17] treated it when people quitted smoking within one year. The majority of studies began to follow up date of diagnosis [11, 13, 14, 17, 19, 21], others started after diagnosis [12, 15, 16, 18] and intervals ranged from 2 months to 2 years.

**Table 1:** Characteristics of included studies regarding smoking and mortality in breast cancer survivors

Author/Publication year	Study location	Sample size	Mean follow-up (years)	Death Causes and Cases	Smoking status	Breast Cancer Ascertainment	Adjustment Factors
Passarelli et al. 2016	USA.	4562	11^a^	Overall Deaths:988 CVD Deaths:258 Breast Cancer Specific Deaths:246	Current	Medical Records	Age at diagnosis, study phase, state of residence, and stage at diagnosis and adjusted for education, BMI, parous status, age at first birth, menopausal status, use of postmenopausal hormone therapy, mammography history, alcohol consumption, first-degree family history of breast cancer, post-diagnosis BMI, post-diagnosis alcohol consumption, and time from diagnosis to post-diagnosis questionnaire.
Nechuta et al. 2016	USA. China.	WHEL:211LACE:1543NHS:2935 All:6596	WHEL:13.6^a^; LACE:12.6^a^; NHS:10.5^a^; All:12.0^a^;	Overall Deaths:1427; WHEL:374; LACE:387; NHS:666;	Former	Cancer Registration	Age at diagnosis, TNM stage, PR status, chemotherapy, radiotherapy, surgery, hormonal therapy, race/ethnicity, menopausal status, co morbidity (diabetes, hypertension), other studied lifestyle factors (as appropriate) and time between exposure measurement and 5-year post diagnosis date, stratified by study. Models for weight change also adjusted for diagnosis BMI.
Kakugawa et al. 2015	Japanese.	848	6.7^a^	All-cause Deaths:170 Breast Cancer Specific Deaths:132;	Ever	Cancer Registration	Age, BMI, stage, hormone receptor, radiation therapy, chemotherapy, endocrine therapy, family history of breast cancer in father, mother, brother or sister, physical activity, co-morbidities, menopausal status and passive smoking from spouse.
Izano et al. 2015	USA.	975	11^a^	Other-Cause Deaths:436; Breast Cancer Specific Deaths:317;	Current	Histological Biopsy	Age at breast cancer diagnosis, breast cancer treatment, race/ethnicity, BMI, financial adequacy, education, positive lymph node involvement, tumor size at diagnosis, co morbidity.
Boone et al. 2015	USA.	2218	10.6^a^	All-cause Deaths:445; Breast Cancer Specific Deaths:243;	Current Ever	Histological Biopsy	Age, study, ethnicity, TMN stage, BMI, alcohol consumption, and education
Seibold et al. 2014	Germany.	3340	6^a^	All-cause Deaths:418; Other-cause Deaths:115; Breast Cancer Specific Deaths:303;	Current	Cancer Registration	Age at diagnosis and study region, adjustment for tumor size, nodal status, metastasis status, histological grading, joint ER and PR, mode of detection, radiotherapy, adult BMI, hormone replacement use at diagnosis, alcohol consumption, CVD, diabetes.
Pierce et al. 2014	USA.	9975	11.1^a^	Overall Deaths:1803; Breast Deaths:1059;	Former	Medical Records	Age at diagnosis, cancer stage, TNM grade, race/ethnicity, education, and obesity.
Bérubé et al. 2014	Canada.	5892	7	All-cause Deaths:1408; Other-cause Deaths:441; Breast Cancer Specific Deaths:953; Unknown-cause Deaths:14;	Current	Doctor-patient Interview	Age at diagnosis, year of diagnosis, age at menarche, parity, menopausal status, current hormone replacement therapy use, first degree family history of breast cancer, ER and PR positive, histological grade, size of the tumor, regional or distant involvement, regional treatment, neo adjuvant therapy, adjuvant endocrine therapy, adjuvant chemotherapy.
Saquib et al. 2013	USA.	2593	7.3	All-cause Deaths:297; Breast Cancer Specific Deaths:245;	Ever	Cancer Registration	Age, cancer stage, tumor grade, ER at diagnosis; chemotherapy and tamoxifen use post-diagnosis; race, obesity, education, menopausal status, alcohol consumption, physical and mental health at trial entry; time between diagnosis and study entry.
Dal Maso et al. 2008	Italy.	1453	10.76	Overall Deaths:503; Breast Deaths:398;	Current	Histological Biopsy	Region of residence, age at diagnosis, year of diagnosis, TNM stage and ER/PR status.
Sagiv et al. 2007	USA.	1273	5.56	All-cause Deaths:188; Breast Cancer Specific Deaths:111;	Current Ever	Doctor-patient Interview	Age at diagnosis, household income, hormone-replacement therapy, and length of residence in interview home.

Abbreviations: BMI, body mass index; WHEL: Women's Healthy Eating & Living Study; LACE: Life After Cancer Epidemiology Study; NHS, Nurses' Health Study; TNM, tumor node metastasis; ER, estrogen receptor; PR, progesterone receptor; CVD, cardiovascular disease.

a Median value.

The risk estimates were mostly adjusted for age, cancer stage and therapies. Subjects with diagnosed BC were ascertained by cancer registration [13, 14, 17, 19], pathological biopsy [15, 16, 20], medical records [12, 18], and doctor-patient interview [11, 21]. The measurement of smoking dimensions was obtained by questionnaires in all the eligible studies. The information regarding status and causes of mortality was mainly obtained from death certificate [13, 17–19, 21] and death registry [15, 16].

As for quality assessment, all 11 studies were found to be of high quality, indicating the quality of included studies was generally good (Supplementary Table S2).

### Association of smoking and risk of BC specific mortality

**Figure 2:**
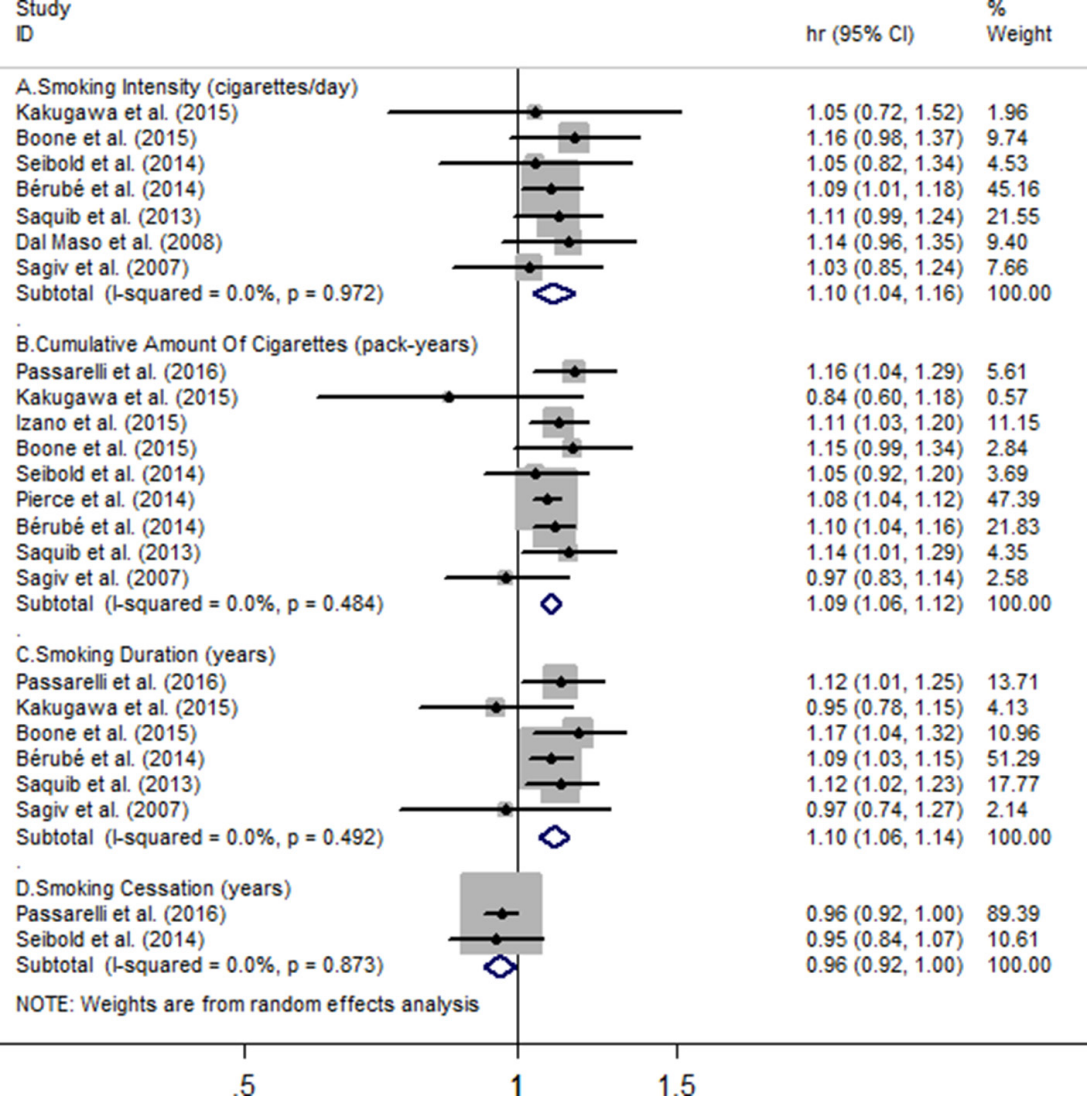
Meta-analysis on (**A**) smoking intensity (cigarettes/day), (**B**) cumulative amount of cigarettes, (**C**) smoking duration, (**D**) smoking cessation and the risk of breast cancer specific mortality. The squares represent the hazard ratio per (A) 10 cigarettes/day, (B) 10 pack-years, (C-D) 10 years increase for each individual study, with the area reflecting the weight assigned to the study. The horizontal line across each square represents the 95% confidence interval. The diamond represents the summary hazard ratio per (A) 10 cigarettes/day, (B) 10 pack-years, (C-D) 10 years increase in smoking doses or duration, with width representing 95% confidence interval.

Seven studies including a sum of 17617 subjects with BC and 2383 deaths were eligible for association between smoking intensity and the risk of BC specific mortality, and pooled HR for every 10 cigarettes/day increment in smoking intensity was 1.10 (95% CI = 1.04 to 1.16), with evidence of no heterogeneity (*I*^2^ = 0%, *P_heterogeneity_* = 0.97) (Figure 2A). Nine individual studies were included in this two-stage meta-analysis of cumulative amount of cigarettes and risk of BC specific mortality involving 31676 BC participants and 3609 deaths, and every 10 pack-years increment in cumulative amount of smoking increased the risk of BC specific mortality (HR = 1.09, 95% CI = 1.06 to 1.12). No heterogeneity was found among these studies (*I*^2^ = 0%, *P_heterogeneity_* = 0.48) (Figure 2B). For the association of smoking duration and BC specific mortality, six studies including 17386 BC cases and 1930 deaths reported it. The summary hazard ratio of BC specific death risk for an increase of every 10 years' smoking was 1.10 (95% CI = 1.06 to 1.14), and low heterogeneity was found (*I*^2^ = 0%, *P_heterogeneity_* = 0.49) (Figure 2C). Only two of included studies revealed the association between smoking cessation and the risk of BC specific risk. Among 7902 BC individuals and 549 deaths, combined hazard ratio for every increase 10 years quitting from smoking is 0.96 (95% CI = 0.92 to 1.00) (Figure 2D), and no evidence of heterogeneity was found (*I*^2^ = 0%, *P_heterogeneity_* = 0.87) (Figure 2C).

### Association of smoking with risk of all-cause mortality

**Figure 3:**
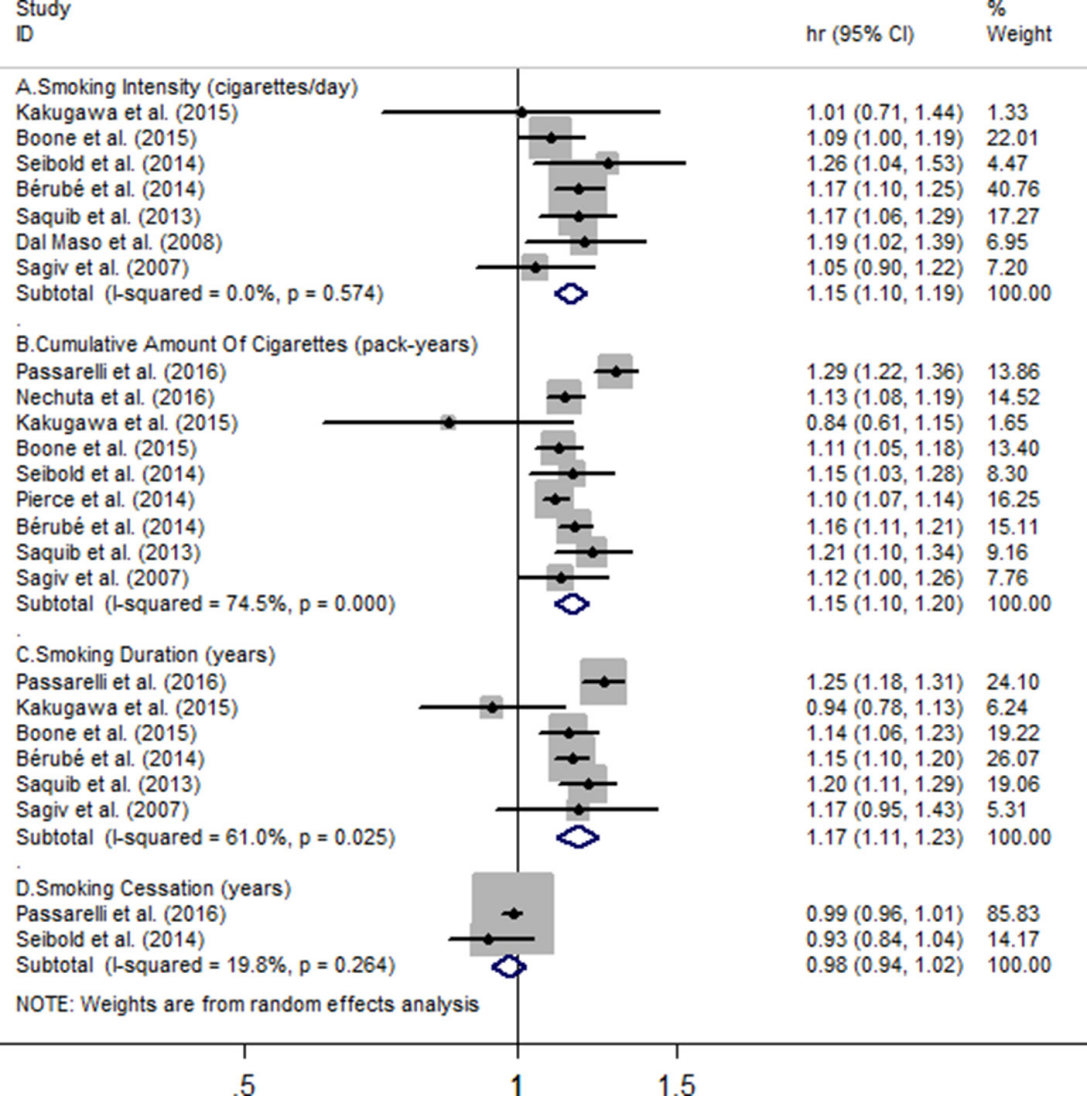
Meta-analysis on (**A**) smoking intensity (cigarettes/day), (**B**) cumulative amount of cigarettes, (**C**) smoking duration, (**D**) smoking cessation and the risk of all-cause mortality. The squares represent the hazard ratio per (A)10 cigarettes/day, (B) 10 pack-years, (C-D) 10 years increase for each individual study, with the area reflecting the weight assigned to the study. The horizontal line across each square represents the 95% confidence interval. The diamond represents the summary hazard ratio per (A) 10 cigarettes/day, (B) 10 pack-years, (C-D) 10 years increase in smoking doses or duration, with width representing 95% confidence interval.

Seven studies were enrolled in the meta-analysis on smoking intensity and risk of all-cause mortality, involving 17617 BC cases and 3429 deaths, and corresponding results showed the for every 10 cigarettes/day increment in smoking intensity, the all-cause mortality risk significantly increased by 15% (HR = 1.15, 95% CI = 1.10 to 1.19), with no heterogeneity (*I*^2^ = 0%, *P_heterogeneity_* = 0.57) (Figure 3A). Nine eligible studies including 37297 BC individuals and 7144 deaths reported the association between cumulative amount of cigarettes and all-cause mortality risk, and every 10 pack-years of cigarettes increment increased the risk of all-cause death (HR = 1.15, 95% CI = 1.10 to 1.20), with substantial heterogeneity (I^2^=74.5%, *P_heterogeneity_* = 0) (Figure 3B). Six studies involving 17386 BC participants and 3506 deaths revealed that every 10 pack-years of cigarettes increment was associated with increased all-cause mortality (HR = 1.17, 95% CI = 1.11 to 1.23), and substantial heterogeneity was found (*I*^2^ = 61%, *P_heterogeneity_* = 0.025) (Figure 3C). Only two studies, among 7902 BC cases and 1406 deaths, the combined hazard ratio of all-cause mortality for an increment of every 10 years quitting smoking is 0.98 (95% CI = 0.94 to 1.02). Heterogeneity of marginal effect estimates on hazard ratio was observed (*P* = 0.26, *I*^2^ = 19.8%) (Figure 3D).

### Linear and non-linear dose-response analyses

**Figure 4:**
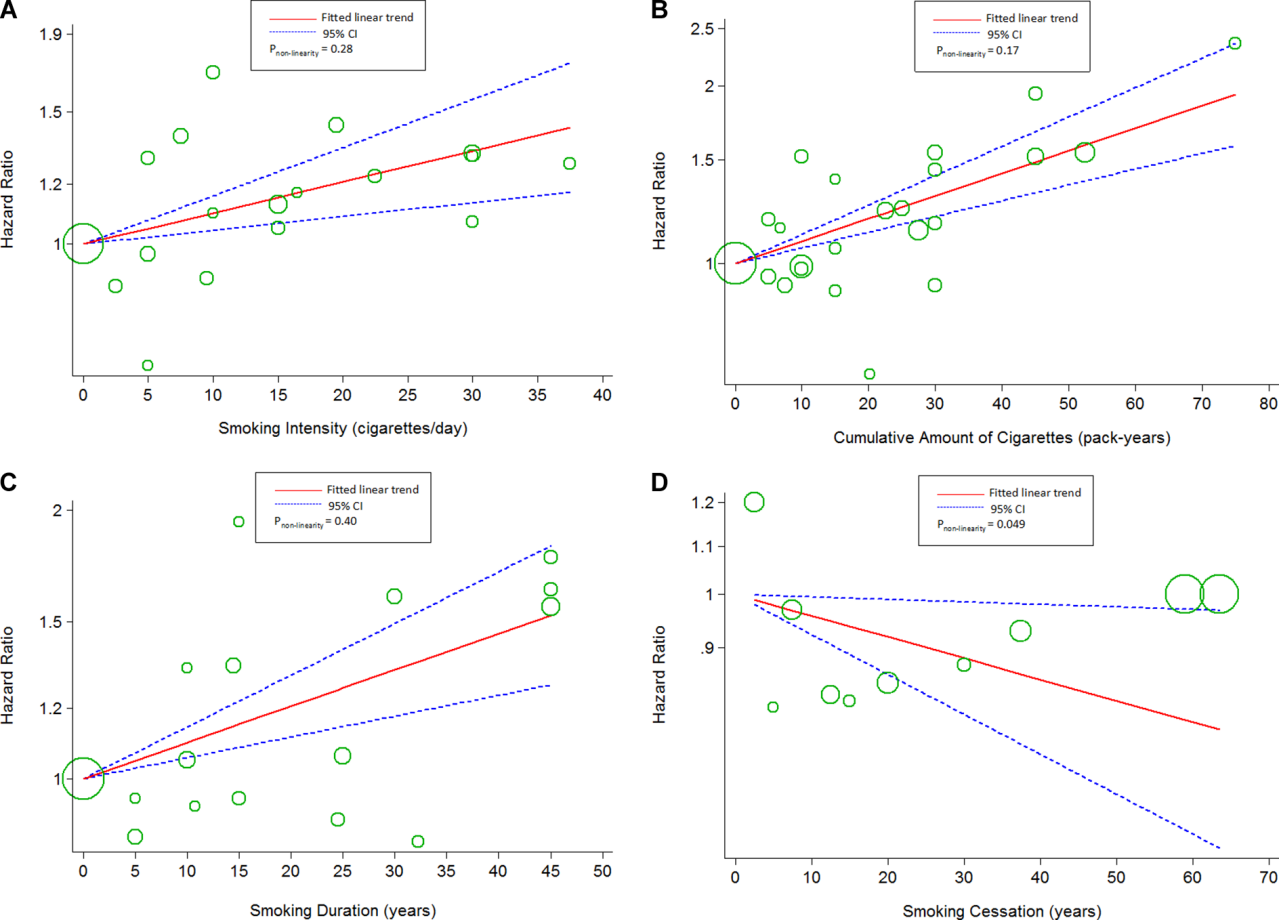
The dose-response analyses on (**A**) smoking intensity (cigarettes/day), (**B**) cumulative amount of cigarettes, (**C**) smoking duration, (**D**) smoking cessation and the risk of breast cancer specific mortality. The circles represent the hazard ratios in each individual study, with the area reflecting the weight assigned to the study.

**Figure 5:**
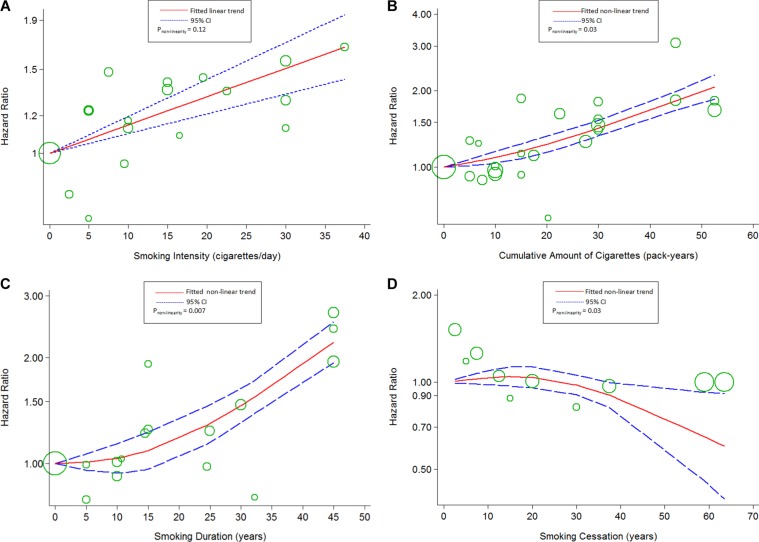
The dose-response analyses on (**A**) smoking intensity (cigarettes/day), (**B**) cumulative amount of cigarettes, (**C**) smoking duration, (**D**) smoking cessation and the risk of all-cause mortality. The circles represent the hazard ratios in each individual study, with the area reflecting the weight assigned to the study.

Using restricted cubic spline function, we found linear associations between smoking intensity (*P_non-linearity_* = 0.28; *chi^2^_model_* = 13.63, *P_model_* = 0.001; Figure 4A), cumulative amount of cigarettes (*P_non-linearity_*=0.17; *chi^2^_model_* = 44.35, *P_model_*< 0.001; Figure 4B), smoking duration (*P_non-linearity_* = 0.40, *chi^2^_model_*=21.94, *P_model_*< 0.001; Figure 4C) and the risk of BC specific mortality. The association between smoking intensity and the risk of all-cause mortality showed a linear curve (*P_non-linearity_* = 0.12; *chi^2^_model_* = 44.2, *P_model_*< 0.001; Figure 5A), too. The evidence of non-linear associations of smoking duration (*P_non-linearity_* = 0.007; *chi^2^_model_* = 138.04, *P_model_*< 0.001; Figure 5C) and the risk of all-cause mortality was found, which was flat when the smoking duration less than 15 years, and rose steeply thereafter. In addition, the linear association of smoking cessation and the risks of BC specific mortality (*P_non-linearity_* = 0.049; *chi^2^_model_*= 4.93, *P_model_*=0.03; Figure 4D) was found, whereas curve in all-cause mortality (*P_non-linearity_*= 0.03; *chi^2^_model_* = 4.79, *P_model_*= 0.09; Figure 5D) were failed to reveal. Only the “marginally” non-linear curve was observed for association between cumulative amount of cigarettes and all-cause death risk (*P_non-linearity_*= 0.03; *chi^2^_model_*= 5.53, *P_model_*= 0.063; Figure 5B), which is likely to a linear curve.

### Subgroup analyses and sensitivity analyses

Various subgroup analyses were conducted to examine the stability of the two-stage meta-analysis's results (Table 2). Except for “sample size < 2000” “former or ever smoking”, “IV stage BC”, “no adjustment for menopausal status” subgroups, the significant associations between an increment of different smoking dimensions and mortality from BC or all causes remained in the rest subgroups (Table 2). Pooled hazard ratios from the random-effects model and fixed-effects model were virtually identical. Omitting a single study in turn did not significantly change the summary risk estimate of either BC or all-cause mortality. Repeating meta-analyses according to various inclusion or exclusion criteria did not change our results, either (Supplementary Table S3).

**Table 2:** Subgroup analyses regarding smoking and mortality in breast cancer survivors between whole studied

Subgroup	BC Specific Mortality					All-Cause Mortality				
	*N*	HR (95% CI)	*I*^2^ (%)	*P*_heterogeneity_	*P*_interaction_	*N*	HR (95% CI)	*I*^2^ (%)	*P*_heterogeneity_	*P*_interaction_
**Sample size^a^**										
**> 2000 [12–19]**										
Intensity(cigarettes/day)^b^	4	1.10 (1.04,1.17)	0	0.90	0.96	4	1.15 (1.10,1.21)	0	0.44	0.78
Cumulative amount (pack-years)^c^	6	1.09 (1.06,1.13)	0	0.72	0.91	7	1.16 (1.11,1.21)	78	0.00	0.57
Duration (years)^d^	4	1.03 (1.02,1.05)	0	0.79	0.88	4	1.05 (1.04,1.07)	58	0.07	0.92
**< 2000 [14–15]**										
Intensity(cigarettes/day)^b^	3	1.09 (0.96,1.22)	0	0.73	-	3	1.11 (1.00,1.23)	0	0.46	-
Cumulative amount (pack-years)^c^	3	1.02 (0.89,1.17)	54	0.12	-	2	1.01 (0.77,1.32)	64	0.10	-
Duration (years)^d^	2	0.98 (0.94,1.03)	0	0.84	-	2	1.01 (0.95,1.08)	62	0.11	-
**Followed- up duration (years)**										
**> 10 [12–16, 18, 20]**										
Intensity(cigarettes/day)^b^	2	1.11 (1.03,1.20)	0	0.33	0.88	2	1.11 (1.03,1.20)	0	0.33	0.88
Cumulative amount (pack-years)^c^	4	1.09 (1.06,1.13)	0	0.54	0.87	4	1.15 (1.08,1.24)	88	0.00	1.00
Duration (years)^d^	2	1.04 (1.02,1.07)	0	0.71	0.76	2	1.06 (1.03,1.09)	77	0.04	0.66
**< 10 [17, 11–14, 21, 15]**										
Intensity(cigarettes /day)^b^	5	1.16 (1.11,1.22)	0	0.55	-	5	1.16 (1.11,1.22)	0	0.55	-
Cumulative amount (pack-years)^c^	5	1.07 (1.01,1.14)	23	0.27	-	5	1.15 (1.10,1.21)	21	0.29	-
Duration (years)^d^	4	1.02 (1.01,1.04)	18	0.30	-	4	1.04 (1.02,1.06)	56	0.08	-
**Smoking status**										
**Current [12, 16, 17, 15, 20–15]**										
Intensity(cigarettes/day)^b^	5	1.10 (1.03,1.16)	0	0.88	0.90	4	1.16 (1.10,1.23)	0	0.47	0.42
Cumulative amount (pack-years)^c^	5	1.11 (1.07,1.15)	0	0.80	0.30	3	1.20 (1.11,1.30)	80	0.01	0.09
Duration (years)^d^	3	1.03 (1.02,1.05)	0	0.61	0.30	2	1.05 (1.03,1.08)	82	0.02	0.53
Intensity(cigarettes/day)^e^	5	1.21 (1.07,1.36)	0	0.88	0.94	4	1.35 (1.21,1.51)	0	0.45	0.44
Cumulative amount (pack-years)^f^	5	1.23 (1.14,1.33)	0	0.79	0.32	3	1.44 (1.23,1.69)	77	0.01	0.11
Duration (years)^g^	3	1.05 (1.03,1.08)	0	0.50	0.56	2	1.09 (1.05,1.14)	84	0.01	0.41
**Ever or former [13, 16, 18, 19, 8, 21]**										
Intensity(cigarettes/day)^b^	2	1.11 (0.99,1.23)	0	0.78	-	3	1.12 (1.05,1.20)	0.0	0.48	-
Cumulative amount (pack-years)^c^	4	1.06 (0.99,1.14)	35	0.20	-	6	1.12 (1.08,1.15)	25	0.24	-
Duration (years)^d^	3	1.01 (0.98,1.05)	30	0.24	-	4	1.04 (1.01,1.07)	55	0.08	-
Intensity(cigarettes/day)^e^	2	1.22 (0.98,1.51)	0	0.78	-	3	1.26 (1.11,1.43)	0	0.48	-
Cumulative amount (pack-years)^f^	4	1.13 (0.98,1.31)	33	0.21	-	6	1.25 (1.18,1.33)	24	0.25	-
Duration (years)^g^	3	1.02 (0.96,1.09)	39	0.19	-	4	1.06 (1.02,1.11)	47	0.13	-
**Tumor stage**										
**I–III [12, 5, 19]**										
Intensity(cigarettes/day)^b^	1	1.11 (0.99,1.24)	/	/	0.97	1	1.17 (1.06,1.29)	/	/	0.88
Cumulative amount (pack-years)^c^	3	1.09 (1.06,1.13)	1.2	0.36	0.99	4	1.18 (1.09,1.27)	89	0.00	0.79
Duration (years)^d^	2	1.03 (1.01,1.06)	0	0.67	0.94	2	1.07 (1.05,1.08)	0	0.54	0.57
**IV [16, 17, 19–20, 15]**										
Intensity(cigarettes/day)^b^	4	1.12 (1.01,125)	0	0.90	-	3	1.13 (1.05,1.21)	0.0	0.44	-
Cumulative amount (pack-years)^c^	4	1.09 (1.02,1.17)	8	0.35	-	3	1.10 (1.01,1.20)	41	0.19	-
Duration (years)^d^	2	1.02 (0.95,1.09)	75	0.05	-	2	1.02 (0.96,1.08)	74	0.05	-
**adjustment for alcohol**										
**Yes [12, 16, 17, 19, 21]**										
Intensity cigarettes/day)^b^	3	1.12 (1.02,1.22)	0	0.80	0.68	3	1.14 (1.07,1.22)	14	0.31	0.80
Cumulative amount (pack-years)^c^	4	1.13 (1.06,1.20)	0	0.69	0.30	5	1.18 (1.10,1.25)	78	0.00	0.27
Duration (years)^d^	3	1.04 (1.02,1.06)	0.0	0.73	0.34	3	1.06 (1.04,1.08)	54.8	0.11	0.24
**No [18, 11, 14–15]**										
Intensity (cigarettes/day)^b^	4	1.09 (1.02,1.16)	0.0	0.89	-	4	1.15 (1.09,1.22)	0.0	0.50	-
Cumulative amount (pack-years)^c^	5	1.08 (1.05,1.20)	15.8	0.31	-	4	1.12 (1.06,1.18)	56.8	0.07	-
Duration (years)^d^	3	1.01 (0.98,1.05)	44.5	0.17	-	3	1.03 (0.99,1.06)	52.8	0.12	-
**adjustment for therapies**										
**Yes [12, 13, 17, 15–14, 21]**										
Intensity (cigarettes/day)^b^	5	1.09 (1.03,1.15)	0.0	0.97	0.84	5	1.16 (1.11,1.22)	0.0	0.55	0.83
Cumulative amount (pack-years)^c^	7	1.10 (1.05,1.14)	9.5	0.36	0.90	7	1.17 (1.11,1.23)	69.4	0	0.40
Duration (years)^d^	5	1.03 (1.02,1.04)	4.3	0.38	0.80	5	1.05 (1.03,1.07)	66.9	0.02	0.67
**No [16, 18, 20]**										
Intensity (cigarettes/day)^b^	2	1.15 (1.02,1.30)	0.0	0.89	-	2	1.11 (1.03,1.20)	0.0	0.33	-
Cumulative amount (pack-years)^c^	2	1.08 (1.05,1.12)	0.0	0.43	-	2	1.10 (1.07,1.13)	0.0	0.79	-
Duration (years)^d^	1	1.05 (1.01,1.09)	/	/	-	1	1.04 (1.02,1.06)	/	/	-
**Adjustment for menopausal status**										
**Yes [12, 13, 15–14]**										
Intensity (cigarettes/day)^b^	3	1.10 (1.03,1.17)	0.0	0.94	0.97	3	1.17 (1.11,1.23)	0.0	0.72	0.97
Cumulative amount (pack-years)^c^	4	1.11 (1.05,1.18)	15	0.32	0.81	5	1.18 (1.10,1.26)	78.6	0.00	0.81
Duration (years)^d^	4	1.03 (1.02,1.04)	9.8	0.34	0.90	4	1.05 (1.02,1.07)	75.2	0.00	0.90
**No [16–18, 19, 20]**										
Intensity (cigarettes/day)^b^	4	1.11 (1.01,1.21)	0.0	0.77	-	4	1.12 (1.05,1.19)	2.5	0.38	-
Cumulative amount (pack-years)^c^	5	1.08 (1.05,1.12)	0.0	0.55	-	4	1.10 (1.07,1.13)	0.0	0.88	-
Duration (years)^d^	2	1.03 (0.98,1.09)	40	0.20	-	2	1.04 (1.02,1.06)	0.0	0.77	-

Abbreviations: N, number of studies; CI, confidence interval; HR, hazard ratio.

a number of BC patients.

b for every 10 cigarettes/day increment.

c for every 10 pack-years increment.

d for every 10 years increment.

e for every 20 cigarettes/day increment.

f for every 20 pack-years increment.

g for every 20 years increment.

-P for interaction is duplicated with corresponding subgroup.

### Publication bias

No evidence of publication bias was found by Begg's test and Egger's test (all *P* > 0.1).

## DISCUSSION

The two-stage dose-response meta-analysis comprising 11 prospective cohort studies suggested that mortality from BC and all causes increased with cigarettes consumption, and marginally decreased with smoking cessation in BC individuals. Linear associations between smoking intensity and the risks of mortality from all causes and BC were revealed, and similar findings on associations of cumulative amount of cigarettes, smoking duration and the risk of BC specific death in BC survivors emerged. Meanwhile, the non-linear associations between cumulative amount of cigarettes, smoking duration and the risk of all-cause mortality in subjects with BC were found, respectively.

A previous meta-analysis [11] indicated that increased risk of BC specific death was observed in BC survivors at current smoking status. The evidence of high heterogeneity was found among their eligible studies, and the inconsistence doses of cigarettes in each category of smoking status most possibly contributed to this heterogeneity. We concentrated on the association of smoking doses or duration and risks of mortality from BC and all causes to fill the gap by calculating a pooled hazard ratio with 95% confidence interval for an increased intake of 10 cigarettes/day or 10 pack-years or 10 years' duration for each study. It is convenient for this expression form that the estimates can be replaced by any other amounts [23]. The approximate 10% risk of BC specific death increased for every increment of 10 cigarettes/day, 10 pack-years, and 10 years' inhalation, respectively, for which risks from all causes nearly increased to 15%. Pack-years of smoking were calculated by multiplying the duration of smoking and the mean number of cigarettes smoked per day divided by 20. The consistent effect on increment of quantity and duration in three smoking dimensions and the risks from all causes and BC reflected the persistence and stability of smoking in included BC patients. The non-linear curve on association of smoking duration and BC specific mortality risk showed relatively flat before 15 pack-years, and rose steeply after it. It was possibly explained that long time smoking was associated with increased mortality from other causes, such as lung cancer [24], cardiovascular events [25], cervical cancer [26], and so on [27].

The link between smoking and the risk of BC and it's specific death has attracted considerable attention. Two previous meta-analyses [11, 22] indicated that compared to non-smokers, the increased risks of BC specific mortality in BC survivors and BC incidence showed among current smokers. Our result according to the association of smoking duration and the BC specific risk in BC survivors shows a more steeply linear curve than that of association between smoking duration and the risk of BC incidence founded by the meta-analysis [22]. There are some plausible mechanisms accounting for these associations. On the one hand, many biological studies support the theory that cigarettes smoking can play an important part in BC carcinogenesis, which not only contain over 60 known carcinogens [28], but also can facilitate this process by disrupting endocrine [29], generating adducts in the breast tissue DNA [30], even transferring the normal human breast epithelial cells *in vitro* [31]. On the other hand, cigarettes smoked can further increase the metastatic ability of BC cells [32], resistant the BC therapies [33], and interact with genetic variation among BC survivors through a series of potential signaling pathways [34].

Although the low or no heterogeneity existed in most associations between smoking and the risk of mortality from BC or all causes, stratified analyses were also conducted to explore the potential effect modifiers. Of the studies with BC patient less than 2000, we found no significant association between smoking and the risks of BC death and all-cause deaths. Considering limited participants and relatively wide CIs for risk estimates, the failure to detect significant associations was possibly caused by lack of power. In respect to group of “IV stage” and “no adjustment for menopausal”, only marginally significant associations between smoking duration and the risks of BC specific death and all-cause death were found. The 5-year relative survival rate for women diagnosed with localized breast cancer is 98.6%; for those with regional and distant-stage breast cancer, the survival rate declines to 84.4% and 24.3%, respectively [35]. Additionally, a study [29] suggested cigarettes smoked exert a dual action on the breast, with different effects in premenopausal and postmenopausal women. Thus, the tumor stage and the status of menstruation are possibly potential modifiers.

A number of epidemiological studies without dose hierarchy suggested no increased risk for former or ever smoking among BC survivors for either all-cause mortality or BC specific mortality [11, 12, 15, 21, 36]. However, Saquib.et.al suggested that the lifetime smoking exposure often referring to cumulative doses of cigarettes, rather than smoking status, should be used to assess mortality risk in former smokers [19], and several studies [13, 14, 16, 18, 19] consistently indicated that former smokers with 20 pack-years or over had significantly higher risks of both mortality from BC or all causes. However, different results were obtained in our subgroup analyses (Table 2). Comparing with current smokers of BC survivors, ever or former smoking only increased all-causes mortality risk not only in low levels of smoking intensity (10 cigarettes/day), cumulative of cigarettes (10 pack-years), and duration (10 years), but also further increased the risk in higher levels (20 cigarettes/day, 20 pack-years, 20years). Nevertheless, the estimates of smoking cessation and the risks of mortality from BC and all causes were marginally significant, perhaps due to insufficient data. Maybe it means that when BC patients inhaled great number of cigarettes in the past, these survivors were still related to a increased risks of all-cause mortality even quitting from smoking after diagnosing.

Although no direct evidence of benefit for BC patients quitting smoking is found, emerging studies and our two-stage meta-analysis consistently support the guidelines from ACS BC Survivorship Care, and non-smokers tend to have better health and live longer than the smokers in BC survivors. To realize a comprehensive surveillance for BC patients, stopping smoking should be suggested by clinicians.

The notable strength of the two-stage dose-response meta-analysis was to clarify the associations and its' shapes between smoking intensity, cumulative amount of cigarettes, smoking duration, smoking cessation and the risk of BC specific and all-cause mortality, and to the best of our knowledge, some clinical implications for understanding the dose-response curves for smoking and disease-specific mortality were proposed, such as liver cancer [37], prostate cancer [38],cardiovascular events [25], leaving a blank in BC, we are the first to explore theses. The consumption of cigarettes in studies varies from each other, and a recent meta-analysis [11] use hazard ratio for “current smokers” versus “non-smokers” to increasing the potential bias. We performed the dose-response meta-analysis to realize a low-heterogeneity model, and various subgroup analyses and sensitivity analyses were conducted to certify the stable outcomes. Prospective cohort studies have the advantage of less biases for selection and recall than case–control studies. Additionally, most studies included in our study had adjusted major confounders, such as age, tumor stage, therapies, alcohol intake and so on. Multiple studies published in recent 1 or 2 years have been appended to our meta-analysis in an attempt to update and validate the associations.

The strengths of this meta-analysis are clear, but some limitations of our study should be acknowledged. Firstly, we never attempt to search unpublished studies, which may lead to missing relevant studies. Then, on account of the limited studies and samples, we cannot calculate the statistically significant summary results for associations of smoking cessation and the risks of all-cause and BC death. In addition, the bias in the accuracy of measurement to smoking is inevitable, because the exposure of cigarette was self-reported by questionnaires rather than reflected by biological markers [39]. Some eligible studies used death certificate to ascertain the causes of death, which is inaccurate in some conditions [40]. Importantly, included studies are all observational, our results might be affected by the potential confounding factors. However, the effect of these confounders on the study outcomes is likely to limited by employing adjusted risk estimates with 95% CI and conducting various subgroup analyses. Lastly, although we cannot find any publication bias in Begg's test and Egger's test, the publication bias must exist in this meta-analysis.

## CONCLUSIONS

Higher smoking intensity, more cumulative amount of cigarettes consumption and longer time for smoking is associated with elevated risk of mortality from BC and all causes in BC individuals. On account of limited samples, smoking cessation among BC patients marginally reduce the risks of BC specific and all-cause mortality. Subgroup analyses suggest a positive association between ever or former smoking and the risk of all-cause mortality in BC patients. Prospective cohort studies with larger sample sizes and longer follow-up times are warranted to further clear the relationships between smoking cessation and mortality risks and the former smoking-mortality association in BC survivors.

## MATERIALS AND METHODS

### Search strategy

A systematic literature search of PubMed and EMBASE databases was conducted through March 2016. The search strategy in detail is exhibited in the Supplementary List S1. We conducted a manual search of reference lists of included studies and current reviews. No attempt was made to identify unpublished reports. If necessary, the original authors were contacted to obtain extra information via e-mails. This meta-analysis was performed in adherence to PRISMA statement [41].

### Study selection

Two investigators (F.L. and Z.Y.L.) independently selected the suitable publications according to following inclusion criteria: (1) Participants: subjects with diagnosed BC. (2) Exposure: different quantities of smoking, including intensity (cigarettes/day), cumulative amount of cigarettes (pack-years), duration (years) and cessation (years). (3) Outcome: Adjusted risk estimates with 95% confidence interval (CI) for at least three quantitative smoking categories on the associations of smoking and the risks of death from all causes or BC. (4) Extra data: the number of death cases, the total subjects or person-years. (4) study design: prospective cohort studies.

### Data extraction and quality assessment

Data extraction was performed by one investigator (F.L.), and was then checked independently for the accuracy by another investigator (X.Z.). The following information was extracted: first author, publication year, study location, sample size, death causes and cases, mean follow-up duration, smoking status, BC assessment and maximally adjusted risk estimate with corresponding 95% confidence interval (CI) and adjustment factors.

Two investigators (F.L. and X.Z.) assessed the quality of included studies independently using the Newcastle-Ottawa quality assessment scale [42]. After evaluating its 3 aspects (selection, comparability, and outcome), nine stars could be assigned to each study at most (4 stars for selection; 3 stars for comparability; 2 stars for outcome).The quality of studies ranks as low quality (below 3 stars), moderate quality (4–6 stars), high quality (7–9 stars). Any disagreements on the results of data extraction and quality assessment were resolved by further discussion.

### Data synthesis and analysis

In this meta-analysis, hazard ratio (HR) with 95% confidence intervals (CI) was considered as a common measure of the association between smoking and the risks of mortality from all causes and BC. The HRs were extracted from “current” or “ever” status of smoking except two studies [20, 21], which were supplied from “former” status. Four classification criteria of smoking were included as follows: intensity (cigarettes/day), cumulative amount of cigarettes (pack-years), duration (years) and cessation (years). Owing to the distinct cut off points for smoking categories in different studies, we calculated HRs with 95% CIs for every 10 cigarettes/day, 10 pack-years, 10 years' smoking, 10 years since quitting smoking increment in cigarettes consumption or smoking duration, respectively. A random-effects model [43] was used to pool HRs to obtain the overall the effect size. The between-study heterogeneity were assessed by Q statistic (*P_Heterogeneit_* < 0.10 suggesting statistically significance) and the I^2^ statistic [44], with following criteria for explanation: high heterogeneity, *I*^2^ > = 75%; moderate heterogeneity, 50.0% < *I*^2^ < 75%; low heterogeneity, *I*^2^ < 50% [45].

A two-stage dose-response meta-analysis was conducted to assess whether the more consumption of cigarettes were associated with higher risks of mortality from all causes and BC based on specific smoking doses or duration, distribution of death cases and person years, and adjusted HRs with 95% CIs. Firstly, we use the generalized least square regression described by Orsini and colleagues [46] to calculate the specific-study linear trend and 95% CIs for every 10 cigarettes per day, 10 pack-years, 10 years' smoking, 10 years since quitting smoking increment in cigarettes consumption or smoking duration within each study from the natural logs of adjusted HRs and CIs across the categories of cigarettes doses. Then, the random-effects model was used to obtain pooled HRs and 95% CIs. The method requires the distribution of cases, person years and the HRs with 95% CIs for at least three quantitative categories of smoking doses or duration. Most original researchers did not report person years by exposure level [11–13, 15–21], and we approximately derived such data from follow-up duration and the number of participants at each smoking level. One study did not provide the number of death cases in every dose category, and we used the relative risks comparing the higher versus lowest categories of smoking to obtain a summary estimate [47]. We designated the midpoint of lower and upper boundaries as the assigned dose if available data parameters of cigarettes doses and duration were reported as range. Furthermore, if the highest category was open ended, the midpoint of the category was set at 1.5 times the lower boundary. When the lowest category was open-ended, we set the lower boundary to zero. We evaluated a potential curve linear association between smoking and risks of mortality from all causes and BC, using restricted cubic splines with three knots at percentiles 10%, 50%, and 90% of the distribution [48, 49]. A *chi^2^_model_* with *P_model_* was calculated to test the suitability of the model. A *P_non-linearity_* value for curve linearity or non linearity was calculated by testing the null hypothesis that the estimated value of the second spline is equal to zero [49].

To identify the potential modifiers, the study-level subgroup analysis was performed stratified by sample size, follow-up duration, smoking status, tumor stage, and adjustment for alcohol, therapies, menopausal status, respectively, and a *P_interaction_* between subgroups was calculated by meta-regression [50]. To further analysis the heterogeneity between eligible studies and check the stability of the pooled results, three sensitive analyses were conducted as following: ignoring a single study in turn; repeating analyses by the fixed-effects model; and employing various eligibility criteria.

Potential publication bias was assessed by Begg rank correlation test [51] and Egger linear regression test [52]. All analyses were conducted using STATA version12.0 (StataCorp, College Station, TX). *P* < 0.05 was considered statistically significant. All statistical tests were two-sided.

## SUPPLEMENTARY MATERIALS






