# Supplementary material for: Smoking increases risks of all-cause and breast cancer specific mortality in breast cancer individuals: a dose-response meta-analysis of prospective cohort studies involving 39725 breast cancer cases
Source: Oncotarget. 2016 Nov 15;7(50):83134–47. doi: 10.18632/oncotarget.13366 (PMC5347758; doi:10.18632/oncotarget.13366)
Supplement: Supplementary file 1 [file oncotarget-07-83134-s001.pdf]

Smoking increases risks of all-cause and breast cancer specific mortality in breast cancer individuals: a dose-response meta-analysis of prospective cohort studies involving 39725 breast cancer cases

Supplementary Materials

Supplementary List S1: Search Strategy

Pubmed

#1: ((((((Smoke [Title/Abstract]) OR Smoking [Title/Abstract]) OR Tobacco [Title/Abstract]) OR Cigarette [Title/Abstract]) OR Cigarettes [Title/Abstract]) OR Nicotine [Title/Abstract]) OR Baccy [Title/Abstract]

#2: (Breast [Title/Abstract]) OR Mammary [Title/Abstract]

#3: ((((Cancer [Title/Abstract]) OR Neoplasm [Title/Abstract]) OR Tumors [Title/Abstract]) OR Malignancy [Title/Abstract]) OR Carcinoma [Title/Abstract]

#4: #2 AND #3

#5: (Death [Title/Abstract]) OR Mortality [Title/Abstract]

#6: #1 AND #4 AND #5

#7: (review[Publication Type]) OR letter[Publication Type]

#8: #6 NOT #7

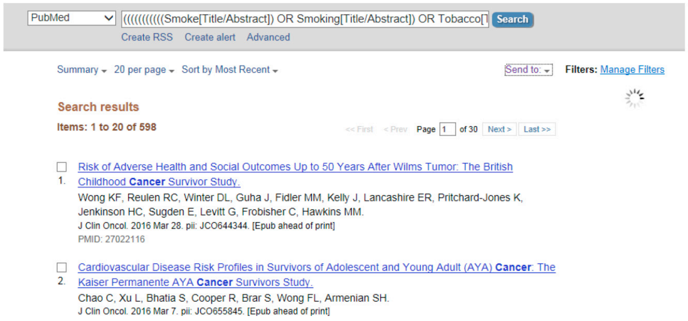

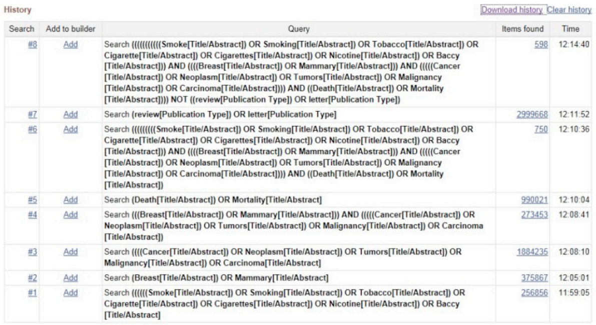

EMBASE

#1: ‘death’:ab,ti AND ([article]/lim OR [article in press]/lim OR [conference abstract]/lim OR [conference paper]/lim) AND [embase]/lim

#2: ‘mortality’:ab,ti AND ([article]/lim OR [article in press]/lim OR [conference abstract]/lim OR [conference paper]/lim) AND [embase]/lim

#3: #1 OR #2

#4: ‘smoke’:ab,ti AND ([article]/lim OR [article in press]/lim OR [conference abstract]/lim OR [conference paper]/lim) AND [embase]/lim

#5: ‘smoking’:ab,ti AND ([article]/lim OR [article in press]/lim OR [conference abstract]/lim OR [conference paper]/lim) AND [embase]/lim

#6: ‘tobacco’:ab,ti AND ([article]/lim OR [article in press]/lim OR [conference abstract]/lim OR [conference paper]/lim) AND [embase]/lim

#7: ‘cigarette’:ab,ti AND ([article]/lim OR [article in press]/lim OR [conference abstract]/lim OR [conference paper]/lim) AND [embase]/lim

#8: ‘Nicotine’:ab,ti AND ([article]/lim OR [article in press]/lim OR [conference abstract]/lim OR [conference paper]/lim) AND [embase]/lim

#9: #4 OR #5 OR #6 OR #7 OR #8

#10: ‘breast’:ab,ti AND ([article]/lim OR [article in press]/lim OR [conference abstract]/lim OR [conference paper]/lim) AND [embase]/lim

#11: ‘mammary’:ab,ti AND ([article]/lim OR [article in press]/lim OR [conference abstract]/lim OR [conference paper]/lim) AND [embase]/lim

#12: #10 AND #11

#13: ‘cancer’:ab,ti AND ([article]/lim OR [article in press]/lim OR [conference abstract]/lim OR [conference paper]/lim) AND [embase]/lim

#14: ‘neoplasm’:ab,ti AND ([article]/lim OR [article in press]/lim OR [conference abstract]/lim OR [conference paper]/lim) AND [embase]/lim

#15: ‘tumors’:ab,ti AND ([article]/lim OR [article in press]/lim OR [conference abstract]/lim OR [conference paper]/lim) AND [embase]/lim

#16: ‘malignancy’:ab,ti AND ([article]/lim OR [article in press]/lim OR [conference abstract]/lim OR [conference paper]/lim) AND [embase]/lim

#17: 'carcinoma':ab,ti AND ([article]/lim OR [article in press]/lim OR [conference abstract]/lim OR [conference paper]/lim) AND [embase]/lim

#18: #13 OR #14 OR #15 OR #16 OR #17

#19: #12 AND #18

#20: #3 AND #9 AND #19.

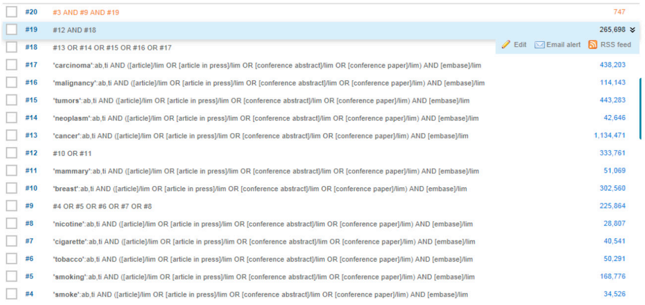

#20	#3 AND #9 AND #19	747
#19	#12 AND #18	265,698
#18	#13 OR #14 OR #15 OR #16 OR #17	
#17	'carcinoma':ab,ti AND ([article]/lim OR [article in press]/lim OR [conference abstract]/lim OR [conference paper]/lim) AND [embase]/lim	438,203
#16	'malignancy':ab,ti AND ([article]/lim OR [article in press]/lim OR [conference abstract]/lim OR [conference paper]/lim) AND [embase]/lim	114,143
#15	'tumors':ab,ti AND ([article]/lim OR [article in press]/lim OR [conference abstract]/lim OR [conference paper]/lim) AND [embase]/lim	443,283
#14	'neoplasm':ab,ti AND ([article]/lim OR [article in press]/lim OR [conference abstract]/lim OR [conference paper]/lim) AND [embase]/lim	42,646
#13	'cancer':ab,ti AND ([article]/lim OR [article in press]/lim OR [conference abstract]/lim OR [conference paper]/lim) AND [embase]/lim	1,134,471
#12	#10 OR #11	333,761
#11	'mammary':ab,ti AND ([article]/lim OR [article in press]/lim OR [conference abstract]/lim OR [conference paper]/lim) AND [embase]/lim	51,069
#10	'breast':ab,ti AND ([article]/lim OR [article in press]/lim OR [conference abstract]/lim OR [conference paper]/lim) AND [embase]/lim	302,560
#9	#4 OR #5 OR #6 OR #7 OR #8	225,864
#8	'nicotine':ab,ti AND ([article]/lim OR [article in press]/lim OR [conference abstract]/lim OR [conference paper]/lim) AND [embase]/lim	28,807
#7	'cigarette':ab,ti AND ([article]/lim OR [article in press]/lim OR [conference abstract]/lim OR [conference paper]/lim) AND [embase]/lim	40,541
#6	'tobacco':ab,ti AND ([article]/lim OR [article in press]/lim OR [conference abstract]/lim OR [conference paper]/lim) AND [embase]/lim	50,291
#5	'smoking':ab,ti AND ([article]/lim OR [article in press]/lim OR [conference abstract]/lim OR [conference paper]/lim) AND [embase]/lim	168,776
#4	'smoke':ab,ti AND ([article]/lim OR [article in press]/lim OR [conference abstract]/lim OR [conference paper]/lim) AND [embase]/lim	34,526

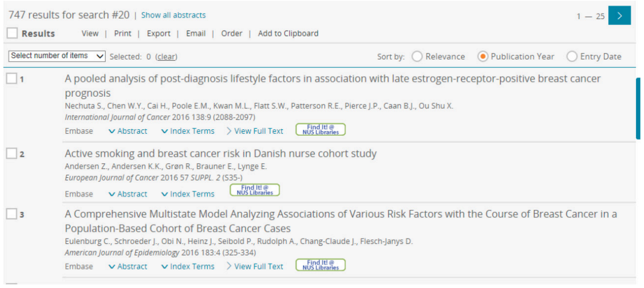

Supplementary Table S1: Exclusion reasons. See Supplementary_Table_S1

Supplementary Table S2: Results of quality assessment

	Selection[1]				Comparability[2]		Outcome[3]			
Source (reference)	Representativeness of exposed cohort ★	Selection of non-exposed cohort ★	Exposure ascertainment ★	No death when investigatio-ns begin ★	Comparable on confounder[5] ★ ★		Outcome Assessment ★	Adequate follow-up (≧10y) ★	Loss to follow-up rate(≦20%) ★	Total Score[4]
Passarelli, et al.	★	★	★	★	★		★	★		7
Nechuta, et al.		★	★	★	★	★	★	★		8
Kakugawa, et al.	★	★	★	★	★	★	★	★	★	9
Izano, et al.		★	★	★	★	★	★	★	★	8
Boone, et al.	★	★		★	★	★	★	★	★	7
Seibold, et al.	★	★	★	★	★	★	★	★	★	9
Pierce, et al.	★	★	★	★	★		★	★		6
Bérubé, et al.	★	★	★	★	★	★	★	★		8
Saquib, et al.		★	★	★	★	★	★	★	★	8
Dal Maso, et al.		★	★	★	★		★	★		6
Sagiv, et al.	★	★	★	★	★		★	★		7

[1]"Selection" part includes representativeness of cases, selection of controls, exposure ascertainment, and no death when investigation begin.
[2]"Comparability" part includes comparable on confounders.
[3]"Outcome" part includes outcome assessment, adequate follow-up, and loss to follow-up rate.
[4]The total score is equal to the total number of stars.
[5]If the studies adjusted for two individual factors among age, therapy, stage, it is award a star. If the studies adjusted for two lifestyle factors among body mass index, physical activity, and alcohol consumption, it is award another star.

Supplementary Table S3: Sensitivity analyses regarding smoking and mortality in breast individuals. See Supplementary_Table_S3
