# Supplementary material for: Smoking increases risks of all-cause and breast cancer specific mortality in breast cancer individuals: a dose-response meta-analysis of prospective cohort studies involving 39725 breast cancer cases
Source: Oncotarget. 2016 Nov 15;7(50):83134–47. doi: 10.18632/oncotarget.13366 (PMC5347758; doi:10.18632/oncotarget.13366)
Supplement: Supplementary file 2 [file oncotarget-07-83134-s002.docx]

**S**u**pplementary Table S1 Exclusion reasons**

| No. | Title | Year | First author | Journal | Reason |
| --- | --- | --- | --- | --- | --- |
| 1 | Overall survival of breast cancer patients in relation to preclinically determined total serum cholesterol, body mass index, height and cigarette smoking: A population-based study | 1991 | Vatten | European Journal of Cancer | Without HR |
| 2 | Breast cancer in Denmark. Incidence, risk factors, and characteristics of survival | 1993 | Ewertz | Acta oncologica | Case-control study |
| 3 | Cancer. Mortality trends for selected smoking-related cancers and breast cancer, 1950-1990 | 1994 | John | Weekly epidemiological record | Cross-Sectional Study |
| 4 | Cigarette smoking and risk of fatal breast cancer | 1994 | Calle | American Journal of Epidemiology | Participants with cancer-free population |
| 5 | Smoking history and cancer patient survival: a hospital cancer registry study | 1997 | Yu | Cancer detection and prevention | Subjects without breast cancer |
| 6 | Family environment, hobbies and habits as psychosocial predictors of survival for surgically treated patients with breast cancer | 1998 | Tominaga | Japanese journal of clinical oncology | Outcome without at least three quantitative smoking categories |
| 7 | Survival of women with breast cancer in relation to smoking | 2000 | Manjer | European Journal of Surgery | Outcome without at least three quantitative smoking categories |
| 8 | Mortality from breast carcinoma among US women: the role and implications of socio-economics, heterogeneous insurance, screening mammography, and geography | 2003 | Okunade | Health care management science | Review |
| 9 | Smoking and prognosis in women with breast cancer | 2005 | Fentiman | International Journal of Clinical Practice | Subjects without breast cancer |
| 10 | Smoking and survival after breast cancer diagnosis | 2007 | Holmes | International Journal of Cancer | Outcome without at least three quantitative smoking categories |
| 11 | An ecologic study of cancer mortality rates in Spain with respect to indices of solar UVB irradiance and smoking | 2007 | Grant | International Journal of Cancer | Ecologic study |
| 12 | Smoking and mortality in the Japan Collaborative Cohort Study for Evaluation of Cancer (JACC) | 2007 | Ozasa | Asian Pacific journal of cancer prevention | Case-control study |
| 13 | Risk factors for the incidence of breast cancer: Do they affect survival from the disease? | 2008 | Barnett | Journal of clinical oncology : official journal of the American Society of Clinical Oncology | Outcome without at least three quantitative smoking categories |
| 14 | Smoking and survival after breast cancer diagnosis: Role of molecular subtype, body mass index, and menopausal status | 2010 | Braithwaite | Cancer Prevention Research | Duplicate report |
| 15 | Modifiable risk factors and survival in women diagnosed with primary breast cancer: Results from a prospective cohort study | 2010 | Hellmann | European Journal of Cancer Prevention | Outcome without at least three quantitative smoking categories |
| 16 | Smoking at time of breast cancer diagnosis and survival | 2011 | Brisson | American Journal of Epidemiology | Meeting |
| 17 | Prospective structured smoking behavior assessment at diagnosis and long-term cancer survival | 2011 | Warren | Journal of Clinical Oncology | Duplicate report |
| 18 | Long lasting effects of smoking: Breast cancer survivors' inflammatory responses to acute stress differ by smoking history | 2012 | Bennett | Brain, Behavior, and Immunity | Subjects without breast cancer |
| 19 | Dietcomplyf study - A multi-centre uk study on breast cancer - What are the dietary and lifestyle changes following diagnosis? | 2012 | Perkins | European Journal of Cancer | Review |
| 20 | The California Breast Cancer Survivorship Consortium (CBCSC): Prognostic factors associated with racial/ethnic differences in breast cancer survival | 2012 | Vigen | Cancer Prevention Research | Case-control study |
| 21 | Smoking and survival after breast cancer diagnosis: A prospective observational study and systematic review | 2012 | Braithwaite | Breast Cancer Research and Treatment | Outcome without at least three quantitative smoking categories |
| 22 | Smoking at diagnosis and survival in cancer patients | 2013 | Warren | International Journal of Cancer | Outcome without at least three quantitative smoking categories |
| 23 | Health behavior in cancer survivorship: Prospective investigations in the danish diet, cancer and health cohort | 2013 | Bidstrup | Psycho-Oncology | Participants including cancer patients and cancer-free population |
| 24 | Lifestyle factors are associated with late breast cancer outcomes among 5-year survivors of estrogen-receptor positive breast cancer | 2014 | Nechuta | Cancer Research | Meeting |
| 25 | Smoking history in relation to survival after a breast cancer diagnosis | 2014 | Newcomb | Cancer Epidemiology Biomarkers and Prevention | Meeting |
| 26 | Influence of metabolic indicators, smoking, alcohol and socioeconomic position on mortality after breast cancer | 2015 | Larsen | Acta oncologica | Outcome without at least three quantitative smoking categories |
| 27 | Post-diagnosis social networks, and lifestyle and treatment factors in the After Breast Cancer Pooling Project | 2016 | Kroenke | Psychooncology | Review |
| 28 | A Comprehensive Multistate Model Analyzing Associations of Various Risk Factors with the Course of Breast Cancer in a Population-Based Cohort of Breast Cancer Cases | 2016 | Eulenburg | American Journal of Epidemiology | Outcome without at least three quantitative smoking categories |

**Abbreviations: HR, hazard ratio.**
