# Supplementary material for: Smoking increases risks of all-cause and breast cancer specific mortality in breast cancer individuals: a dose-response meta-analysis of prospective cohort studies involving 39725 breast cancer cases
Source: Oncotarget. 2016 Nov 15;7(50):83134–47. doi: 10.18632/oncotarget.13366 (PMC5347758; doi:10.18632/oncotarget.13366)
Supplement: Supplementary file 3 [file oncotarget-07-83134-s003.docx]

**S**u**pplementary Table S3 Sensitivity analyses regarding smoking and mortality in breast individuals.**

|  |  | | |  | | |
| --- | --- | --- | --- | --- | --- | --- |
|  | **N** | **HR (95%CI)** | **I^2^ (%)** | **N** | **HR (95%CI)** | **I^2^ (%)** |
| **Random-effects model** |  |  |  |  |  |  |
| Intensity(cigarettes/day)^a^ | 7 | 1.10(1.04,1.16) | 0 | 7 | 1.15(1.10,1.20) | 0 |
| Cumulative amount (pack-years)^b^ | 9 | 1.09(1.06,1.12) | 0 | 9 | 1.15(1.10,1.20) | 75 |
| Duration (years)^c^ | 6 | 1.10(1.06,1.14) | 0 | 6 | 1.17(1.11,1.23) | 61 |
| cessation(year)^c^ | 2 | 0.96(0.92,1.00) | 0 | 2 | 0.98(0.94,1.02) | 20 |
| **Fixed-effects model** |  |  |  |  |  |  |
| Intensity(cigarettes/day)^a^ | 7 | 1.10(1.04,1.16) | 0 | 7 | 1.15(1.10,1.20) | 0 |
| Cumulative amount (pack-years)^b^ | 9 | 1.09(1.06,1.12) | 0 | 9 | 1.14(1.12,1.16) | 75 |
| Duration (years)^c^ | 6 | 1.10(1.06,1.14) | 5 | 6 | 1.18(1.15,1.21) | 61 |
| cessation(year)^c^ | 2 | 0.96(0.92,1.0) | 0 | 2 | 0.99(0.96,1.01) | 20 |
| **Analyses within** | | | | | | |
| **Defined smoking status after breast cancer diagnosis.** |  |  |  |  |  |  |
| Intensity(cigarettes/day)^a^ | 4 | 1.09(1.02,1.17) | 0 | 4 | 1.13(1.07,1.19) | 3 |
| Cumulative amount (pack-years)^b^ | 7 | 1.09(1.06,1.13) | 10 | 7 | 1.14(1.09,1.20) | 80 |
| Duration (years)^c^ | 5 | 1.09(1.04,1.15) | 5 | 5 | 1.16(1.08,1.23) | 68 |
| cessation(year)c  **Studies where non-smoker refer to persons with never smoking.** | 1 | 0.96(0.92,1.00) | / | 1 | 0.99(0.96,1.01) | / |
| Intensity(cigarettes/day)^a^ | 6 | 1.10(1.03,1.16) | 0 | 6 | 1.14(1.09,1.19) | 0 |
| Cumulative amount (pack-years)^b^ | 8 | 1.09(1.06,1.12) | 0 | 8 | 1.14(1.09,1.20) | 77 |
| Duration (years)^c^ | 5 | 1.09(1.04,1.15) | 5 | 5 | 1.16(1.08,1.23) | 68 |

| cessation(year)^c^ | 2 | 0.96(0.92,1.0) | 0 | 2 | 0.98(0.94,1.02) | 20 |
| --- | --- | --- | --- | --- | --- | --- |
| **Adjustment for both age at diagnoses, therapies and tumor stage.** |  |  |  |  |  |  |
| Intensity(cigarettes/day)^a^ | 4 | 1.09(1.03,1.16) | 0 | 4 | 1.17(1.11,1.23) | 0 |
| Cumulative amount (pack-years)^b^ | 6 | 1.11(1.07,1.15) | 0 | 6 | 1.18(1.11,1.25) | 73 |
| Duration (years)^c^ | 4 | 1.09(1.05,1.14) | 0 | 4 | 1.17(1.09,1.25) | 75 |
| cessation(year)^c^ | 2 | 0.96(0.92,1.0) | 0 | 2 | 0.98(0.94,1.02) | 20 |
| **Analyses without** | | | | | | |
| **Sample size** ^d^ **>6000** |  |  |  |  |  |  |
| Intensity(cigarettes/day)^a^ | 7 | 1.10(1.04,1.16) | 0 | 7 | 1.15(1.10,1.20) | 0 |
| Cumulative amount (pack-years)^b^ | 8 | 1.10(1.06,1.14) | 0 | 7 | 1.16(1.10,1.23) | 71 |
| Duration (years)^c^ | 6 | 1.10(1.06,1.14) | 0 | 6 | 1.17(1.11,1.23) | 61 |
| cessation(year)^c^ | 2 | 0.96(0.92,1.0) | 0 | 2 | 0.98(0.94,1.02) | 20 |
| **Sample size** ^d^ **<1000** |  |  |  |  |  |  |
| Intensity(cigarettes/day)^a^ | 6 | 1.10(1.04,1.16) | 0 | 6 | 1.15(1.10,1.20) | 0 |
| Cumulative amount (pack-years)^b^ | 7 | 1.09(1.06,1.12) | 0 | 7 | 1.16(1.11,1.21) | 75 |
| Duration (years)^c^ | 5 | 1.11(1.06,1.05) | 0 | 5 | 1.19(1.14,1.23) | 43 |
| cessation(year)^c^ | 2 | 0.96(0.92,1.0) | 0 | 2 | 0.98(0.94,1.02) | 20 |
| **Former smoking** |  |  |  |  |  |  |
| Intensity(cigarettes/day)^a^ | 7 | 1.10(1.04,1.16) | 0 | 7 | 1.15(1.10,1.20) | 0 |
| Cumulative amount (pack-years)^b^ | 8 | 1.10(1.06,1.14) | 0 | 7 | 1.16(1.10,1.23) | 71 |
| Duration (years)^c^ | 6 | 1.10(1.06,1.14) | 0 | 6 | 1.17(1.11,1.23) | 61 |
| cessation(year)^c^ | 2 | 0.96(0.92,1.0) | 0 | 2 | 0.98(0.94,1.02) | 20 |

**Abbreviations: N, number of studies; CI, confidence interval; HR, hazard ratio.**

**^a^ for every 10 cigarettes/day increment**

**^b^ for every 10 pack-years increment**

**^c^ for every 10 years increment**

^d^ **number of breast cancer patients**
